# Dengue Myocarditis: A Retrospective Study From 2019 to 2023 in a Tertiary Care Hospital in Bangladesh

**DOI:** 10.7759/cureus.84371

**Published:** 2025-05-18

**Authors:** Sumaiya Farah Marisa, Nikhat Shahla Afsar, Chowdhury Adnan Sami, Md. Imrul Hasan, Nishat Ali, Mohammad Mahfuzur Rahman, Sayat Quayum Mohona, Rayhan Uddin Ahmed, Anik Ehsan, Faisal Baset

**Affiliations:** 1 Internal Medicine, Evercare Hospital Dhaka, Dhaka, BGD; 2 Internal Medicine, Bangabandhu Sheikh Mujib Medical University, Dhaka, BGD

**Keywords:** bangladesh, clinical profile, dengue, mortality, myocarditis, risk factor

## Abstract

Background

A major global health problem is dengue illness, which is rising in Bangladesh between 2019 and 2023. Dengue infections can present with a wide range of symptoms, from fever to severe organ involvement like myocarditis. In this retrospective analysis, risk factors, clinic-laboratory profile, and outcomes of dengue myocarditis in hospitalised patients were investigated.

Methods

The study was a retrospective one, conducted in a tertiary private hospital in Dhaka between 2019 and 2023. Dengue infection was diagnosed by positive dengue nonstructural antigen (NS1) tests and/or dengue IgM or dengue RT-PCR (reverse transcription polymerase chain reaction) positivity. Dengue myocarditis was diagnosed with the European Society of Cardiology (ESC) 2013 consensus statement. For each myocarditis patient, we recruited age-, gender-, and year-of-admission-matched control dengue patients.

Results

Thirty-four dengue-myocarditis patients and 81 matched dengue patients were recruited as controls, with the mean age of myocarditis patients being 48.5 ± 20.1 years; 20.7% were from the 60-69 age group, and 21 (61.7%) were male. Mean (±standard deviation) leucocyte count 9.1 (±4.7) ×10^9^/L, alanine aminotransferase (ALT) 604 (±937) IU/L, aspartate aminotransferase (AST) 2076 (±4202) IU/L, and C-reactive protein (CRP) 5.5 (±5.3) mg/dL were significantly higher in the myocarditis group. Among 34 myocarditis patients, four patients (11.7%) died, with procalcitonin 18.8 (±27.5) ng/mL, prothrombin time (PT) 28.7 (±12.4) seconds, and activated partial thromboplastin time (APTT) 102.6 (±46.9) seconds being significantly higher. Multivariable logistic regression showed that the presence of comorbidities (odds ratio [OR]: 10.5; 95% confidence interval [CI]: 2.78-39.5]) and a raised neutrophil-lymphocyte ratio (NLR) (OR 11.9; 95% CI: 2.82-50.7) were significantly associated with myocarditis.

Conclusion

The presence of comorbidities and a higher NLR ratio were significantly associated with developing dengue myocarditis.

## Introduction

Dengue virus is the cause of dengue infection, which belongs to the genus Flavivirus within the Flaviviridae family. Any of the four dengue virus serotypes (DENV) spread by Aedes mosquitoes might result in infection [1]. Recently, dengue in Bangladesh has been on the rise, as the Directorate General of Health Services reported 514,537 dengue cases and 2,273 fatalities between 2019 and 2023 [2].

Clinically, dengue infections can range from mild fever to severe dengue that includes substantial bleeding, shock, plasma leakage, and multi-organ failure along with severe thrombocytopenia. Myocarditis, an inflammatory condition of the cardiac muscle, has received increased attention in recent years as one of the less common but potentially fatal dengue complications [3]. Data are scarce regarding this devastating complication of dengue from Southeast Asia, especially from Bangladesh.

The various cardiovascular manifestations of dengue infection are explained by the complex pathophysiology. Initially, direct cardiac tropism and viral replication are the ability of the dengue virus to invade and replicate within cardiac cells directly, as seen with other viruses. Secondly, immune response and cytokine storm: Patients with dengue fever have higher levels of serum tumour necrosis factor and interleukins 6, 13, and 18, which enhance vascular permeability and cause shock. The unchecked inflammatory cascade exacerbates myocardial inflammation and injury, damaging heart tissues [4]. Predicting length of stay and in-hospital mortality is significantly influenced by cardiac problems during the illness. Its pathophysiology is complicated and includes direct viral invasion of cardiac tissues, immune-mediated pathogenic pathways, and the host immune system's reaction to the infection [5]. From modestly elevated cardiac enzymes to severe myocarditis, dengue fever's cardiac symptoms can result in arrhythmias, congestive heart failure, cardiogenic shock, and even death [5].

Despite the growing recognition of dengue myocarditis as a severe complication, data on key outcomes such as ICU admission, mortality, and prognostic biochemical markers (e.g., procalcitonin, prothrombin time [PT], activated partial thromboplastin time [APTT]) remain limited, particularly in low-resource settings. This gap hampers early risk assessment and targeted management in endemic regions like Bangladesh. In this retrospective analysis, we analysed the risk factors, clinic-laboratory profile, and outcomes of dengue myocarditis in hospitalised patients.

## Materials and methods

Study design, setting, and participants

This single-centre, retrospective study was done in dengue patients who were admitted to the medical ward at a tertiary private hospital in Bangladesh, which is situated in the country's capital, Dhaka, between 2019 and 2023. 

**Figure 1:**
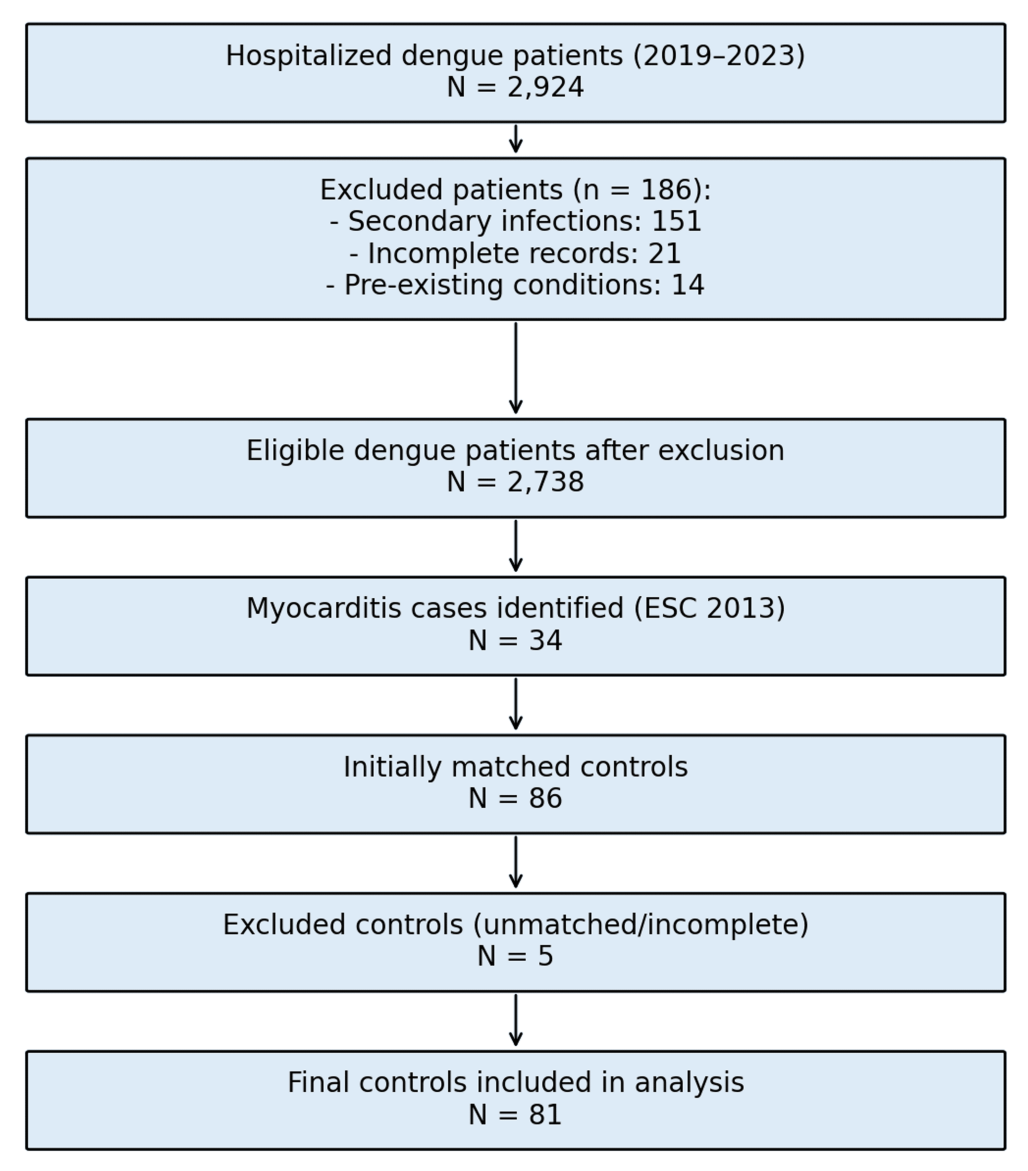
STROBE flow diagram of patient inclusion, exclusion, and matching ESC: European Society of Cardiology.

Inclusion criteria were hospitalised patients ≥12 years old with laboratory-confirmed dengue (positive dengue NS1 antigen, positive dengue IgM antibody, or positive dengue RT-PCR [reverse transcription polymerase chain reaction]). Patients in the myocarditis group had to fulfil the 2013 European Society of Cardiology (ESC) diagnostic criteria of myocarditis with at least one clinical feature (unexplained palpitation, new or worsening breathlessness, or unexplained shock) and one diagnostic criterion (elevated troponin I, with or without ECG changes) [6]. The troponin I normal level was 15 pg/mL in our laboratory. Controls were dengue hospitalised patients without clinical or laboratory evidence of myocarditis and were matched to the cases by age (±5 years), sex, and year of hospital admission. Patients who developed acute coronary syndrome during the disease course, those who had proven secondary infection (bacterial sepsis), pre-existing hematologic malignancy, chronic liver disease/cirrhosis, platelet disorders (idiopathic thrombocytopenic purpura), incomplete medical records, and those who were transferred out to other centers before the treatment was completed were excluded. For each myocarditis patient, we tried to recruit age-, gender-, and year-of-admission-matched control dengue patients. But, due to limitations in record availability and strict matching criteria (age, gender, year of admission), we ultimately recruited 81 control dengue patients. A patient flow diagram in STROBE style is given in Figure 1.

According to the World Health Organization's 2009 categorisation, dengue severity was divided into three groups based on discharge or death: Group A, Group B, and Group C [7]. Patients with dengue fever without warning signs are in Group A; those with warning signals are in Group B; and those with severe dengue fever (severe plasma leakage, severe haemorrhage, severe organ involvement) are in Group C. Group C is designated as the severe group, whereas Groups A and B are merged into the non-severe group. Myocarditis patients corresponded to Group C, while controls corresponded to Groups A and B, according to the WHO 2009 classification.

Study procedure

After obtaining IRB clearance, data like demographics, age, gender, length of illness, hospitalisation days, symptoms, and dengue fever type and laboratory indicators were obtained using a structured questionnaire from patient records available at the Medical Record Department (MRD) of Evercare hospital.

After selecting all the dengue cases from the MRD server, relevant information will be collected in a preformed questionnaire according to inclusion-exclusion criteria. Data was extracted by four licensed physician doctors from internal medicine departments who were pre-trained by the principal investigator. 

Ethics statement

This retrospective study was conducted in compliance with the World Medical Association's (WMA) Declaration of Helsinki. The study protocol was examined and approved by Evercare Hospital Dhaka's Department of Ethical Review Committee (approval number ERC 50/2024-02). Data for the survey was solely gathered from the medical records of dengue patients who were admitted to this hospital, and the patients' anonymity was strictly maintained.

Statistical analyses

SPSS (version 28.0; IBM Corp, Armonk, NY) was used for statistical analysis. The chi-square or Fisher's exact tests for numerical variables and independent t-tests, if applicable for continuous variables, were used to compare clinical and laboratory data between groups. To find the risk factors for myocarditis, multivariable logistic regression was used. Any test with a p-value below 0.05 was deemed statistically significant.

## Results

Baseline characteristics of the patients

We retrospectively recruited 34 myocarditis patient profiles. To compare, we also recruited age-, gender-, and year-matched controls in a 1:2 ratio. Among 115 patients who were selected, the mean age was 47.2 ± 18.4 years, and 55.6% were male patients. 87.8% of patients were residents of Dhaka city.

According to the ESC 2013 criteria, dengue fever with myocarditis was diagnosed in 34 (29.6%) patients, and most of them were male patients (61.7%). Age and gender were matched in the non-severe control group (81 out of 115 patients). A large number of participants were between the ages of 60 and 69 (22.6%), including myocarditis cases (20.5%).

Comorbidities were more frequent in the myocarditis group (79.4%) compared to the control group (38.3%) (p < 0.001). The most common comorbidity was hypertension in the myocarditis group, affecting 61.7% (p = 0.003), followed by diabetes, which was 38.2% (p = 0.037); 11.7% had chronic pulmonary disease (p = 0.04), and 14.7% were obese (p = 0.003).

Out of 115 patients, 99 were positive for dengue NS1 antigen, which was 86% of the total, and 20 patients (17.4%) had positive dengue-specific IgM antibody, and 6.1% had additionally positive anti-dengue IgG antibody (Table 1).

**Table 1:** Baseline characteristics of the patients BA: bronchial asthma; CKD: chronic kidney disease; CAD: coronary artery disease; CLD: chronic liver disease; CPD: chronic pulmonary disease; COPD: chronic obstructive pulmonary disease; DM: diabetes mellitus; HTN: hypertension; CVD: cerebrovascular disease; SD: standard deviation; NS1: non-structural protein 1; Ig: immunoglobulin; Ab: antibody. *Chi-square test was done. **Independent t-test was done.

Characteristics	Total n (%) (N = 115)	Myocarditis n (%) [N = 34 (29.6%)]	Control n (%) [N = 81 (70.4%)]	Chi-square value/t-test value	p-Value
Age (years), mean (±SD)	47.2 (±18.4)	48.5 (±20.1)	46.6 (±17.7)	0.5	0.63**
Age category (years)				0.03	0.835*
12-19	11 (9.6%)	4 (11.7%)	7 (8.6%)		
20-29	7 (6.1%)	2 (5.8%)	5 (6.1%)		
30-39	23 (20%)	5 (14.7%)	18 (22.2%)		
40-49	20 (17.4%)	6 (17.6%)	14 (17.2%)		
50-59	15 (13%)	4 (11.7%)	11 (13.6%)		
60-69	26 (22.6%)	7 (20.5%)	19 (23.45)		
70-79	13 (11.3%)	6 (17.6%)	7 (8.6%)		
Sex				0.4	0.393*
Male	64 (55.6%)	21 (61.7%)	43 (53.1%)		
Residence				4.4	0.016*
Dhaka	101 (87.8%)	26 (76.4%)	75 (92.6%)		
Outside Dhaka	14 (12%)	8 (23.5%)	6 (7.4%)		
Comorbidities (Yes)	58 (50.4%)	27 (79.4%)	31 (38.3%)	10.6	<0.001*
DM	29 (25.2%)	13 (38.2%)	16 (19.7%)	3.4	0.037*
HTN	47 (40.8%)	21 (61.7%)	26 (32.1%)	7.5	0.003*
CAD	12 (10.4%)	5 (14.3%)	7 (8.6%)	0.4	0.33*
CPD (BA/COPD)	6 (5.2%)	4 (11.7%)	2 (2.4%)	2.5	0.04*
CLD	2 (1.7%)	2 (5.8%)	0	2.02	0.16*
CKD	4 (3.4%)	4 (11.7%)	0	6.6	0.001*
Obesity	6 (5.2%)	5 (14.7%)	1 (1.2%)	6.2	0.003*
CVA	2 (1.7%)	1 (2.9%)	1 (1.2%)	-	-
Dengue NS1 antigen					
Positive	99 (86%)	31 (91.1%)	68 (83.9%)	-	-
Anti-dengue IgM Ab					
Positive	20 (17.4%)	4 (11.7%)	16 (19.7%)	-	-
Anti-dengue IgG Ab					
Positive	7 (6.1%)	2 (5.8%)	5 (6.2%)	-	-

Clinical presentations

One hundred percent of the cases had fever, followed by myalgia in 33.9%, weakness in 33%, and headache in 24.3%. Headache was more common in non-severe cases (p = 0.004). Among other symptoms, retro-orbital pain was present in 12.2%, cough in 6.1%, dyspnea in 13%, chest pain in 4.2%, undue tachycardia in 16.5%, loose stool in 17.3%, and anorexia in 17.3% of the patients. In myocarditis, chest pain (p = 0.012), dyspnea (p = 0.001), and undue tachycardia (p < 0.001) were more common. However, bradycardia was noted in three patients, and all of them were non-severe cases.

The most common warning sign was abdominal pain (22.6%), followed by persistent vomiting (21.7%), and mucosal bleeding was present in 6.1% of cases. Haematemesis and melena were 1.7% for each, and haematochezia in 0.8% of cases. Warning signs were more common in myocarditis, but the results were insignificant.

Out of all patients, severe bleeding (2.6%), disseminated intravascular coagulation (4.3%), acute respiratory distress syndrome (1.7%), neurologic involvement (5.2%), and acute hepatitis (7.8%) were documented only in the myocarditis group.

The patients' average length of stay in the hospital was 5.25 (±3.7) days. The control group's mean hospital stay was 3.73 days, while the myocarditis group's was 8.8 days, and the result was significant (p = 0.001). The majority of the patients made a full recovery and were discharged. ICU admission was required for 27 (23.4%) cases, which were all from the myocarditis group, contributing to 79.4% (27 out of 34) of total myocarditis patients. A total of four (3.4%) case fatalities were reported, and all were from the myocarditis group (Table 2).

**Table 2:** Clinical presentations DIC: disseminated intravascular coagulation; ARDS: acute respiratory distress syndrome. *Chi-square test was done. **t-Test was done.

Characteristics	Total n (%), N = 115	Myocarditis n (%), N = 34	Control n (%), N = 81	Chi-square value/t-test value	p-Value
Fever	115 (100%)	34 (100%)	81 (100%)	-	-
Headache	28 (24.3%)	4 (11.7%)	24 (29.6%)	3.24	0.04*
Restlessness	4 (3.5%)	4 (11.7%)	0	-	-
Weakness	38 (33%)	14 (41.2%)	24 (29.6%)	0.97	0.23*
Cough	7 (6.1%)	3 (8.8%)	4 (4.9%)	0.14	0.42*
Retroorbital pain	14 (12.2%)	5 14.7%	9 (11.1%)	0.05	0.59*
Abdominal tenderness	26 (22.6%)	8 (23.5%)	18 (22.2%)	0.02	0.87*
Anorexia	20 (17.3%)	9 (26.4%)	11 (13.5%)	2.1	0.09*
Persistent vomiting	25 (21.7%)	9 (26.4%)	16 (19.7%)	0.3	0.45*
Loose stool	20 (17.3%)	9 (26.4%)	11 (13.5%)	2.1	0.09*
Chest pain	5 (4.2%)	4 (11.7%)	1 (1.2%)	4.1	0.012*
Lethargy	28 (6.9%)	10 (33.3%)	18 (22.2%)	0.3	0.4*
Rapid breathing	15 (13%)	14 (41.2%)	1 (1.2%)	28.1	0.001*
Myalgia	39 (33.9%)	12 (35.2%)	27 (33.3%)	0.02	0.83*
Mucosal bleeding	7 (6.1%)	3 (8.8%)	4 (4.9%)	0.1	0.42*
Undue tachycardia	19 (16.5%)	15 (44.1%)	4 (4.9%)	23.8	<0.001*
Bradycardia	3 (2.6%)	0	3 (3.7%)	-	-
Melena	2 (1.7%)	1 (2.9%)	1 (1.2%)	0.01	0.5*
Haematemesis	2 (1.7%)	1 (2.9%)	1 (1.2%)	0.01	0.5*
Haematochezia	1 (0.8%)	0	1 (1.2%)	-	-
Severe bleeding	3 (2.6%)	3 (8.8%)	0	-	-
DIC	5 (4.3%)	5 (14.7%)	0	-	-
ARDS	2 (1.7%)	2 (5.8%)	0	-	-
Neurologic involvement	6 (5.2%)	6 (17.6%)	0	-	-
Acute hepatitis	9 (7.8%)	9 (26.4%)	0	-	-
Total hospital days, mean (±SD)	5.25 (±3.7)	8.8 (±5.1 )	3.73 (±1.8 )	22.1	0.001**
ICU stay	27 (23.4%)	27 (79.4%)	0	-	-
Death	4 (3.4%)	4 (11.7%)	0	-	-

Haematological, biochemical, and radiological parameters

According to haematologic markers, the myocarditis group had a lower mean haematocrit of 36.9% (±8.39) (p = 0.235) than the total cohort, which had a mean of 37.8% (±5.6). The whole cohort's total leucocyte count (mean ± SD) was 6.9 ×10^9^/L (±3.8), with the myocarditis group having a higher mean of 9.1 × 10^9^/L (±4.7) (p < 0.001). The platelet count's overall mean (±SD) was 77.29 × 10^9^/L (±45.11); the mean was slightly lower in the myocarditis group, but the result was insignificant. The rise of haematocrit >45% was found in 20.5% of myocarditis cases and 4.9% of non-severe cases (p = 0.009).

Leucopenia was more frequently observed in non-severe cases (22.2%) than in the myocarditis group (14.7%) (p = 0.358), and thrombocytopenia was more common in the non-severe group, 83.9% of the patients compared to myocarditis group (73.5%) (p = 0.195); however, none of these findings were significant.

Alanine aminotransferase (ALT), aspartate aminotransferase (AST), and albumin levels were reported in relation to liver function tests. The total participants' mean (±SD) serum ALT was 250 IU/L (±560), and the mean serum AST was 706 (±2432 IU/L). Both the means were higher in the myocarditis group, ALT 604 IU/L (±937) and AST 2076 IU/L (4202) (p < 0.001). Raised serum ALT >45 IU/L and AST >45 IU/L were more frequently observed in dengue myocarditis, 88.2% for both ALT and AST (p = 0.017, p = 0.07, respectively). Mean serum albumin was 3.2 g/dL (±0.4), significantly lower in the myocarditis group (p = 0.003). C-reactive protein (CRP) was also significantly raised in the myocarditis group (5.5 ± 5.3 mg/dL vs 2.3 ± 2.1 mg/dL, p < 0.001). Albuminuria was found in nine (7.9%) cases belonging to the myocarditis group.

Ascites and pleural effusion were the most prevalent findings in the ultrasound screening (USG) profile. In the myocarditis group, 14.3% of the cases had ascites; on the contrary, 11.1% in the control group had ascites; pleural effusion was present in 44.1% in the myocarditis group compared to only 4.9% in the control group (p = 0.002 and 0.001, respectively) (Table 3).

**Table 3:** Haematological, biochemical, and radiological parameters ALT: alanine aminotransferase; AST: aspartate aminotransferase; Hct: haematocrit; SD: standard deviation; USG: ultrasound screening; CRP: C-reactive protein. Leucopenia was defined as WBC <4000 cells/µL; thrombocytopenia as platelet count <150,000/µL. *Student's t-test was done. **Chi-square test was done.

Characteristics	Total (n = 115)	Myocarditis (n = 34)	Control (n = 81)	Chi-square/t-test value	p-Value
Haematological mean (±SD)					
Hct (%)	37.8 (±5.6)	36.9 (±8.39)	38.2 (±3.8)	-0.87	0.235*
Leucocyte count (cells × 10^9^/L)	6.9 (±3.8)	9.1 (±4.7)	5.9 (±2.9)	3.7	<0.001*
Platelet count (cells × 10^9^/L)	77293 (±45116)	76852 (±56740)	77478 (±39653)	-0.06	0.95*
Biochemical parameters, mean (±SD)					
Serum ALT (IU/L)	250 (±560)	604 (±937)	101 (±91)	3.12	<0.001*
Serum AST (IU/L)	706 (±2432)	2076 (±4202)	130 (±115)	2.7	<0.001*
Serum albumin (mg/dL)	3.2 (±0.4)	3.1 (±0.5)	3.3 (±0.4)	-2.1	0.003*
CRP (mg/dL)	3.28 (±3.6)	5.5 (±5.3)	2.3 (±2.1)	3.4	<0.001*
Categorical data, n (%)					
Rise of Hct >45	11 (9.6%)	7 (20.5%)	4 (4.9%)	5.1	0.009**
Leucopenia (<4000)	23 (20%)	5 (14.7%)	18 (22.2%)	0.4	0.358**
Thrombocytopenia (<1,50,000)	93 (80.9%)	25 (14.7%)	68 (83.9%)	1.1	0.195**
Serum ALT [>45 (IU/L)]	84 (73%)	30 (88.2%)	54 (66.6%)	4.6	0.017**
Serum AST [>45 (IU/L)]	88 (77.4%)	30 (88.2%)	59 (72.8%)	2.4	0.07**
Urinary albumin	9 (7.9%)	9 (26.4%)	0	-	-
Fluid leakage (USG)					
Ascites	21 (18.3%)	12 (14.3%)	9 (11.1%)	7.8	0.002**
Pleural effusion	19 (16.5%)	15 (44.1%)	4 (4.9%)	17.1	0.001**

Myocarditis profile

Out of 34 myocarditis cases, four patients died. When we compared the survived patients with the case fatalities, a significant increase was found in procalcitonin, PT, and APTT levels between these two groups (p = 0.046, 0.003, and 0.013, respectively) (Table 4).

**Table 4:** Myocarditis profile APTT: activated partial thromboplastin time; PT: prothrombin time; BNP: brain natriuretic peptide; CRP: C-reactive protein; s: second; mcg: microgram. *Student's t-test was done.

Characteristics, mean (±SD)	Total (n = 34)	Survival (n = 30)	Death (n = 4)	t-Test value	p-Value*
Procalcitonin (ng/mL)	7.4 (±11.9)	5.9 (±7.8)	18.5 (±27.8)	-0.9	0.046
Fibrinogen (mg/dL)	215 (±123)	225.6 (±127)	137.2 (±38.6)	2.9	0.184
APTT (s)	64.6 (±33.4)	59.3 (±28.5)	102.6 (±46.9)	-1.8	0.013
PT (s)	18.1 (±8)	16.6 (±6.2)	28.7 (±12.4)	-1.9	0.003
Troponin I (ng/L)	1728 (±3291)	1899 (±3481)	484 (±266)	2.1	0.43
BNP (pg/mL)	5090 (±6220)	5252 (±6545)	3915 (±3286)	0.6	0.69
D-dimer (mcg/L)	5680 (±6845)	6104 (±7188)	2603 (±1780)	2.2	0.35
CRP (mg/dL)	5.5 (±5.4)	5.7 (±5.6)	4.2 (±3.5)	0.7	0.6
Serum albumin (g/dL)	3.1 (±0.5)	3.1 (±0.5)	3.2 (±0.3)	-0.5	0.8

Myocarditis risk factors

**Figure 2:**
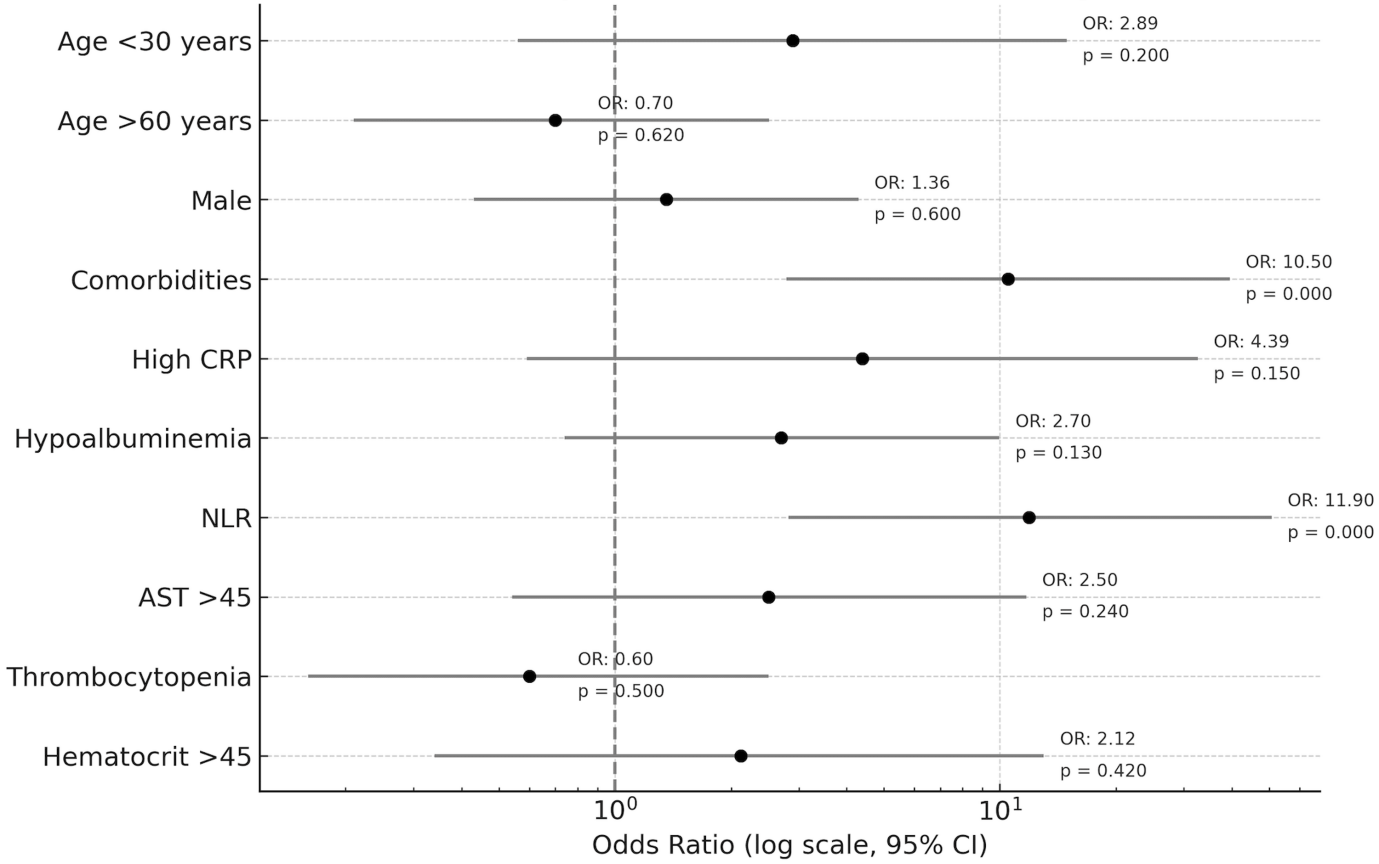
Forest plot of independent risk factors for myocarditis among hospitalised dengue patients The forest plot displays the ORs and 95% CIs for clinical and laboratory variables associated with dengue-related myocarditis, derived from logistic regression analysis. The x-axis is plotted on a logarithmic scale to accurately reflect the multiplicative nature of ORs. The vertical dashed line represents the null effect (OR = 1.0). OR: odds ratio; CI: confidence interval; CRP: C-reactive protein; NLR: neutrophil-lymphocyte ratio; AST: aspartate aminotransferase.

We observed that patients with myocarditis who were infected with dengue had more comorbidities (odds ratio [OR] = 10.5; 95% confidence interval [CI]: 2.78-39.5) and raised neutrophil-lymphocyte ratio (NLR) (OR = 11.9; 95% CI: 2.82-50.7) and both the findings were highly significant with a p-value <0.001. Myocarditis patients were also more likely to have high CRP (OR = 4.39), hypoalbuminemia (OR = 2.7), AST >45 (OR = 2.5), and haematocrit >45 (OR = 2.12), but these findings were not significant (Table 5, Figure 2). 

**Table 5:** Myocarditis risk factors AST: aspartate aminotransferase; OR: odds ratio; CI: confidence interval; CRP: C-reactive protein. Haematocrit (%), AST (IU/L). *Multivariable logistic regression test was done.

Characteristics	OR	95% CI	p-Value*
Age <30 years	2.89	0.56-14.9	0.2
Age >60 years	0.7	0.21-2.51	0.62
Male	1.36	0.43-4.3	0.6
Comorbidities	10.5	2.78-39.5	<0.001
High CRP	4.39	0.59-32.6	0.15
Hypoalbuminemia	2.7	0.74-9.96	0.13
Neutrophil-lymphocyte ratio	11.9	2.82-50.7	<0.001
AST >45	2.5	0.54-11.7	0.24
Thrombocytopenia	0.6	0.16-2.5	0.5
Haematocrit >45	2.12	0.34-13.0	0.42

## Discussion

Myocarditis caused by dengue viral infection is a potentially serious and emerging complication that requires close monitoring due to its impact on disease severity and mortality outcomes. The 2009 WHO updated classification now includes myocarditis in the description of severe dengue. However, in the majority of nations where DENV is endemic, screening is lacking; hence, the actual frequency of myocarditis is unknown [1]. This study’s objective was to compare hospitalized patients with non-myocarditis cases and assess the risk factors, clinic-laboratory profiles, and outcomes of dengue myocarditis.

The majority of study participants (22.6%) were between the ages of 60 and 69; in the case of myocarditis, it was 20.5%, and 61.7% of them were male. A study in Sri Lanka revealed similar results, where male respondents were predominant [8]. This could be because, in our society, men are more likely to have better access to healthcare. Second, because they spend more time outside, men can be more vulnerable to outdoor mosquito bites. However, these results are different from those obtained by Oneda et al. in a study conducted in Brazil, where the most common age group of severe dengue cases was 20-39 (38.3%), and cases were female (55.6%) [9]. Another study on myocarditis also revealed that men and those between the ages of 20 and 40 were at greatest risk, despite the underlying cause [10].

Our study demonstrated that patients with comorbidities have a 10.5 times higher chance of developing cardiac involvement in dengue-infected patients. Compared to healthy individuals, those with comorbidities who have dengue fever appear to be more likely to experience sequelae and/or severe dengue [11]. In our study, 79.4% of myocarditis cases had comorbidities compared to 38.3% in non-myocarditis cases, and the result was significant (p < 0.001). Comorbidities, particularly cardiovascular disease, stroke, diabetes, respiratory and renal illness, and advanced age are associated with severe dengue, according to a systematic literature analysis [11].

According to a matched case-control research, severe dengue and hypertension are significantly correlated [12]. Our analysis also revealed similar results (p = 0.003). There are several reasonable theories, but the precise mechanism that connects hypertension to an increased risk of severe dengue is not fully understood. The host immune system is overactivated in both severe dengue and hypertension [13]. A pro-inflammatory condition associated with hypertension has also been connected to vascular endothelium malfunction, which may result in severe dengue symptoms [14]. Additionally, we discovered a strong correlation between DM and CPD with dengue myocarditis (p = 0.037 and 0.04, respectively). Different studies demonstrated that underlying DM was an independent risk factor for developing severe dengue [15,16]. Micro- and macrovascular functions are compromised in uncontrolled diabetes, which may increase plasma leakage and eventually cause severe dengue [17]. A significant association was also found between obesity and dengue myocarditis compared to non-myocarditis cases (p = 0.003). A study carried out in Malaysia revealed similar results, showing that obesity had a significant impact on the severity of the disease [18].

Chest pain, palpitations, pleurisy, irregular pulses, bradycardia, hypotension, pulmonary oedema, and shock-like symptoms are among the many clinical signs that point to cardiac involvement in dengue [19,20]. The most frequent clinical manifestation of dengue myocarditis in the participants in our study was fever (100%), followed by undue tachycardia (44.1%), weakness/fatigue (41.2%), and rapid breathing/dyspnea (41.2%). Highly significant associations were found among undue tachycardia (p < 0.001), rapid breathing (p = 0.001), and chest pain (p = 0.012) in dengue myocarditis patients compared to non-severe dengue cases. The most prevalent clinical manifestations at presentation in a Pakistani investigation of a group of patients with dengue myocarditis were fever (100%), shortness of breath (100%), and lethargy (100%) [21].

An interesting finding was noted in this study: headache was more common in non-severe cases (29.6%) than dengue myocarditis (11.7%). This result was comparable to a meta-analysis, which demonstrated that headache was a protective element against severe dengue (OR: 0.555), indicating that dengue patients who experienced headaches were less likely to have a severe case of the illness [22].

Dengue patients with cardiac involvement stayed longer in the hospital than non-severe cases, and the result was very significant (p = 0.001). According to a Pakistani study, patients’ coagulopathy, acute kidney injury, and advancing age are linked to longer hospital stays [23]. According to a Malaysian study, long-term hospitalization was substantially linked to patients with hypertension, multiple organ dysfunctions, raised alkaline phosphatase, and prolonged PT and aPTT in DHF [24].

Yingying et al. showed in another study of 201 cases with dengue myocarditis in China that 24% had only positive cardiac biomarkers, and ECG and biomarker changes were both positive in 22.58% of cases, while ECG and biomarker changes were both positive in 21% of cases. They also found that roughly 30% had ECG or echocardiographic changes without elevated cardiac biomarkers [5]. Although troponin-I is a sensitive biomarker of myocyte injury in patients with clinically suspected myocarditis, they are non-specific and, when expected, does not exclude myocarditis, although in our study, all the patients had increased levels of troponin I [25].

Our study revealed that high serum ALT and AST levels are associated with dengue myocarditis, and the result was highly significant compared to non-myocarditis cases (p < 0.001). This result was comparable to other studies that revealed that the rise in AST was more prominent than in ALT levels in patients with severe dengue [26].

Raised CRP and leucocyte counts are also associated with dengue myocarditis, and the results are highly significant compared to non-myocarditis cases (p < 0.001). All inflammatory diseases and tissue damage result in the synthesis of CRP, an acute-phase protein. The liver produces CRP within 6 hours of the onset of inflammation. According to several studies, CRP levels may be a useful biomarker for predicting the severity of dengue in adults [27]. Our study also demonstrated that the NLR is significantly raised in myocarditis (OR: 11.9, p < 0.001). Mirna et al. also reported that NLR was markedly increased in patients with viral myocarditis [28].

Among 34 myocarditis patients, four patients died. According to our research, elevated levels of procalcitonin, PT, and APTT were substantially linked to higher mortality (p = 0.046, 0.003, and 0.013, respectively). Previous studies also collaborated with our findings of raised procalcitonin related to profound shock and higher PT and aPTT associated with a higher risk of mortality [23,29].

There are limitations in our study. First, it is a single-center, retrospective study conducted at a tertiary hospital in Dhaka, Bangladesh, which may limit the generalizability of the findings. The diagnostic criteria for myocarditis were based on clinical symptoms (such as unexplained shock and shortness of breath), which may be subjective and prone to variability, especially in a retrospective data analysis. Also, not all the patients underwent echocardiography, which might have given additional diagnostic accuracy. Furthermore, the gold standard diagnostic test for myocarditis, cardiac MRI, was not done here. We also didn't estimate the frequency of cardiac complications like cardiogenic shock, arrhythmia profile, or vasopressor needs. 

## Conclusions

Dengue has a wide range of presentations, and myocarditis is one of the fatal ones. We found that patients with myocarditis had increased haematocrit, AST, and CRP. Procalcitonin, PT, and APTT values were significantly greater in the group with fatal myocarditis. Those with comorbidities and a higher NLR ratio were more likely to develop dengue myocarditis. This study can serve as a basis for considering possible biomarkers to look into predicting severe cardiac complications in dengue.
